# Supplementary material for: Effect of Isolation Conditions on Diversity of Endolichenic Fungal Communities from a Foliose Lichen, Parmotrema tinctorum
Source: J Fungi (Basel). 2021 Apr 26;7(5):335. doi: 10.3390/jof7050335 (PMC8146534; doi:10.3390/jof7050335)
Supplement: Supplementary file 1 [file jof-07-00335-s001.zip › jof-1163889-supplementary.pdf]

Journal of Fungi

MDPI

Supplementary Materials: Effect of Isolation Conditions on Diversity of Endolichenic Fungal Communities from a Foliose Lichen, *Parmotrema tinctorum*

Table S1. Taxonomic information of isolated ELF species.

No.	Species Name	NI[1]	GenBank No.[2]	Isolation No.[3]	Class	Order	Family
1	*Nigrospora oryzae* complex	72	MT586876	KoLRI49200	Sordariomycetes	Tricho-sphaeriales	Trichosphaeriaceae
2	*Nigrospora lacticolonia* complex	35	MT586877	KoLRI48122	Sordariomycetes	Tricho-sphaeriales	Trichosphaeriaceae
3	*Pestalotiopsis paeoniicola* complex	8	MT586878	KoLRI49369	Sordariomycetes	Xylariales	Sporocadaceae
4	*Robillarda sessilis*	5	MT586879	KoLRI49492	Sordariomycetes	Xylariales	Sporocadaceae
5	*Pestalotiopsis disseminata* complex	26	MT586880	KoLRI48579	Sordariomycetes	Xylariales	Sporocadaceae
6	*Virgaria nigra*	5	MT586881	KoLRI48583	Sordariomycetes	Xylariales	Xylariaceae
7	*Creosphaeria sassafras*	8	MT586882	KoLRI48039	Sordariomycetes	Xylariales	Xylariaceae
8	*Whalleya microplaca*	2	MT586883	KoLRI49195	Sordariomycetes	Xylariales	Xylariaceae
9	Xylariaceae sp.1	29	MT586884	KoLRI47972	Sordariomycetes	Xylariales	Xylariaceae
10	*Hypoxylon howeanum*	1	MT586885	KoLRI48074	Sordariomycetes	Xylariales	Xylariaceae
11	*Hypoxylon vinosopulvinatum*	57	MT586886	KoLRI47966	Sordariomycetes	Xylariales	Xylariaceae
12	*Daldinia childiae* complex	276	MT586887	KoLRI47976	Sordariomycetes	Xylariales	Xylariaceae
13	*Daldinia eschscholtzii*	2	MT586888	KoLRI49194	Sordariomycetes	Xylariales	Xylariaceae
14	*Hypoxylon monticulosum*	5	MT586889	KoLRI48147	Sordariomycetes	Xylariales	Xylariaceae
15	*Hypoxylon* sp. 1	1	MT586890	KoLRI48032	Sordariomycetes	Xylariales	Xylariaceae
16	*Hypoxylon* sp. 2	1	MT586891	KoLRI48845	Sordariomycetes	Xylariales	Xylariaceae
17	*Hypoxylon lateripigmentum*	1	MT586892	KoLRI48545	Sordariomycetes	Xylariales	Xylariaceae
18	*Hypoxylon rubiginosum*	2	MT586893	KoLRI48816	Sordariomycetes	Xylariales	Xylariaceae
19	*Hypoxylon perforatum*	75	MT586894	KoLRI47961	Sordariomycetes	Xylariales	Xylariaceae
20	Xylariaceae sp.2	1	MT586895	KoLRI49193	Sordariomycetes	Xylariales	Xylariaceae
21	Xylariaceae sp.3	1	MT586896	KoLRI48114	Sordariomycetes	Xylariales	Xylariaceae
22	*Muscodor fengyangensis*	2	MT586897	KoLRI48802	Sordariomycetes	Xylariales	Xylariaceae
23	*Muscodor suthepensis*	5	MT586898	KoLRI48554	Sordariomycetes	Xylariales	Xylariaceae
24	*Nemania plumbea*	3	MT586899	KoLRI49779	Sordariomycetes	Xylariales	Xylariaceae
25	*Rosellinia aquila*	12	MT586900	KoLRI48053	Sordariomycetes	Xylariales	Xylariaceae
26	*Rosellinia limonispora*	3	MT586901	KoLRI48058	Sordariomycetes	Xylariales	Xylariaceae
27	*Xylaria arbuscula* complex	46	MT586902	KoLRI48192	Sordariomycetes	Xylariales	Xylariaceae
28	*Xylaria* sp. 1	1	MT586903	KoLRI49039	Sordariomycetes	Xylariales	Xylariaceae
29	*Xylaria schweinitzii*	1	MT586904	KoLRI48348	Sordariomycetes	Xylariales	Xylariaceae
30	*Nemania* sp. 2	4	MT586905	KoLRI48979	Sordariomycetes	Xylariales	Xylariaceae
31	*Nemania serpens*	5	MT586906	KoLRI49774	Sordariomycetes	Xylariales	Xylariaceae
32	*Nemania primolutea*	21	MT586907	KoLRI49044	Sordariomycetes	Xylariales	Xylariaceae
33	*Nemania* sp. 1	2	MT586908	KoLRI47956	Sordariomycetes	Xylariales	Xylariaceae
34	*Xylaria* sp. 2	1	MT586909	KoLRI47988	Sordariomycetes	Xylariales	Xylariaceae
35	*Xylaria longipes*	3	MT586910	KoLRI48141	Sordariomycetes	Xylariales	Xylariaceae
36	*Nemania abortiva*	45	MT586911	KoLRI48799	Sordariomycetes	Xylariales	Xylariaceae
37	*Nemania diffusa*	88	MT586912	KoLRI48010	Sordariomycetes	Xylariales	Xylariaceae
38	*Ophiognomonia setacea*	4	MT586913	KoLRI48814	Sordariomycetes	Diaporthales	Gnomoniaceae
39	*Biscogniauxia maritima*	5	MT586914	KoLRI48023	Sordariomycetes	Xylariales	Xylariaceae
40	*Biscogniauxia* sp.	8	MT586915	KoLRI49386	Sordariomycetes	Xylariales	Xylariaceae
41	*Biscogniauxia petrensis*	68	MT586916	KoLRI49756	Sordariomycetes	Xylariales	Xylariaceae
42	*Annulohypoxylon annulatum*	3	MT586917	KoLRI48240	Sordariomycetes	Xylariales	Xylariaceae

43	*Annulohypoxylon areolatum*	7	MT586918	KoLRI48140	Sordariomycetes	Xylariales	Xylariaceae
44	*Trichoderma* aff. *songyi*	51	MT586919	KoLRI48167	Sordariomycetes	Hypocreales	Hypocreaceae
45	*Trichoderma* sp.	152	MT586920	KoLRI48677	Sordariomycetes	Hypocreales	Hypocreaceae
46	*Sarocladium kiliense*	248	MT586921	KoLRI48025	Sordariomycetes	Hypocreales	Sarocladiaceae
47	*Acremonium rutilum*	1	MT586922	KoLRI49267	Sordariomycetes	Hypocreales	Hypocreales_Inc[4]
48	*Purpureocillium lilacinum*	2	MT586923	KoLRI48648	Sordariomycetes	Hypocreales	Ophiocordycipitaceae
49	*Tolypocladium tropicale*	1	MT586924	KoLRI49290	Sordariomycetes	Hypocreales	Ophiocordycipitaceae
50	*Tolypocladium sp.*	3	MT586925	KoLRI47959	Sordariomycetes	Hypocreales	Ophiocordycipitaceae
51	*Fusarium* aff. *solani*	6	MT586926	KoLRI48861	Sordariomycetes	Hypocreales	Nectriaceae
52	*Fusarium* aff. *oxysporum*	3	MT586927	KoLRI48700	Sordariomycetes	Hypocreales	Nectriaceae
53	*Fusarium* aff. *graminearum*	12	MT586928	KoLRI49656	Sordariomycetes	Hypocreales	Nectriaceae
54	Microascales sp. 1	46	MT586929	KoLRI47997	Sordariomycetes	Microascales	Microascales_Un[5]
55	Microascales sp. 2	13	MT586930	KoLRI47951	Sordariomycetes	Microascales	Microascales_Un[5]
56	*Coniochaeta boothii*	3	MT586931	KoLRI48800	Sordariomycetes	Coniochaetales	Coniochaetaceae
57	*Coniochaeta velutina*	31	MT586932	KoLRI49003	Sordariomycetes	Coniochaetales	Coniochaetaceae
58	*Phaeoacremonium iranianum*	3	MT586933	KoLRI48511	Sordariomycetes	Togniniales	Togniniaceae
59	*Sordaria fimicola*	22	MT586934	KoLRI48040	Sordariomycetes	Sordariales	Sordariaceae
60	*Phialophora cyclaminis*	2	MT586935	KoLRI49307	Eurotiomycetes	Chaetothyriales	Herpotrichiellaceae
61	Sordariales sp.	2	MT586936	KoLRI49024	Sordariomycetes	Sordariales	Sordariales_Un[5]
62	*Cercophora caudata*	2	MT586937	KoLRI49455	Sordariomycetes	Sordariales	Lasiosphaeriaceae
63	*Subramaniula cuniculorum*	7	MT586938	KoLRI48075	Sordariomycetes	Sordariales	Chaetomiaceae
64	*Chaetomium crispatum*	1	MT586939	KoLRI48346	Sordariomycetes	Sordariales	Chaetomiaceae
65	*Chaetomium convolutum*	7	MT586940	KoLRI48236	Sordariomycetes	Sordariales	Chaetomiaceae
66	*Chaetomium globosum*	37	MT586941	KoLRI47957	Sordariomycetes	Sordariales	Chaetomiaceae
67	*Pithya* aff. *vulgaris*	4	MT586942	KoLRI49105	Pezizomycetes	Pezizales	Sarcoscyphaceae
68	*Toxicocladosporium irritans*	5	MT586943	KoLRI49652	Dothideomycetes	Capnodiales	Cladosporiaceae
69	*Cladosporium* aff. *cladosporioides*	11	MT586944	KoLRI49807	Dothideomycetes	Capnodiales	Cladosporiaceae
70	*Paraconiothyrium brasiliense*	5	MT586945	KoLRI49253	Dothideomycetes	Pleosporales	Didymosphaeriaceae
71	*Pyrenochaetopsis leptospora*	2	MT586946	KoLRI49755	Dothideomycetes	Pleosporales	Cucurbitariaceae
72	*Anteaglonium* sp.	5	MT586947	KoLRI48484	Dothideomycetes	Pleosporales	Anteagloniaceae
73	Tympanidaceae sp.	1	MT586948	KoLRI49292	Leotiomycetes	Helotiales	Tympanidaceae
74	*Exophiala bergeri*	13	MT586949	KoLRI49293	Eurotiomycetes	Chaetothyriales	Herpotrichiellaceae
75	*Exophiala moniliae*	10	MT586950	KoLRI49754	Eurotiomycetes	Chaetothyriales	Herpotrichiellaceae
76	*Penicillium* aff. *steckii*	9	MT586951	KoLRI48389	Eurotiomycetes	Eurotiales	Aspergillaceae
77	*Penicillium* aff. *chrysogenum*	23	MT586952	KoLRI49233	Eurotiomycetes	Eurotiales	Aspergillaceae
78	*Mollisia* sp.	1	MT586953	KoLRI48525	Leotiomycetes	Helotiales	Dermateaceae
79	*Hymenochaete yasudai*	14	MT586954	KoLRI48661	Agaricomycetes	Hymenochaetales	Hymenochaetaceae
80	*Sistotrema brinkmannii*	8	MT586955	KoLRI49787	Agaricomycetes	Cantharellales	Hydnaceae
81	*Peniophora incarnata*	13	MT586956	KoLRI48803	Agaricomycetes	Russulales	Peniophoraceae
82	*Peniophora crassitunicata*	5	MT586957	KoLRI48711	Agaricomycetes	Russulales	Peniophoraceae
83	*Coprinellus radians*	10	MT586958	KoLRI49494	Agaricomycetes	Agaricales	Psathyrellaceae
84	*Trametes versicolor*	14	MT586959	KoLRI49495	Agaricomycetes	Polyporales	Coriolaceae
85	*Lenzites betulinus*	4	MT586960	KoLRI49799	Agaricomycetes	Polyporales	Coriolaceae
86	*Trametes cubensis*	2	MT586961	KoLRI49115	Agaricomycetes	Polyporales	Coriolaceae
87	*Cinereomyces lindbladii*	4	MT586962	KoLRI49788	Agaricomycetes	Corticiales	Corticiaceae
88	*Cerrena zonata*	2	MT586963	KoLRI49782	Agaricomycetes	Polyporales	Polyporaceae
89	*Phlebia acerina*	2	MT586964	KoLRI49457	Agaricomycetes	Corticiales	Corticiaceae
90	*Phlebiopsis peniophoroides*	11	MT586965	KoLRI49028	Agaricomycetes	Polyporales	Phanerochaetaceae
91	*Bjerkandera aduMild*	30	MT586966	KoLRI48137	Agaricomycetes	Polyporales	Phanerochaetaceae
92	*Trametopsis cervina*	4	MT586967	KoLRI49476	Agaricomycetes	Polyporales	Polyporaceae
93	*Emmia lacerata*	1	MT586968	KoLRI48440	Agaricomycetes	Polyporales	Meruliaceae

94	*Irpex lacteus*	18	MT586969	KoLRI48485	Agaricomycetes	Polyporales	Irpicaceae
95	*Cladophialophora* aff. *parmeliae*	18	MT586970	KoLRI49329	Eurotiomycetes	Chaetothyriales	Herpotrichiellaceae
96	*Xylaria* sp. 3	2	MT586971	KoLRI49323	Sordariomycetes	Xylariales	Xylariaceae
97	*Nigrospora pyriformis* complex	2	MT586972	KoLRI48143	Sordariomycetes	Trichosphaeriales	Trichosphaeriaceae
98	*Annulohypoxylon* sp.	1	MT586973	KoLRI49177	Sordariomycetes	Xylariales	Xylariaceae
99	*Annulohypoxylon* aff. *stygium*	8	MT586974	KoLRI48867	Sordariomycetes	Xylariales	Xylariaceae
100	*Coniochaeta gigantospora*	1	MT586975	KoLRI49047	Sordariomycetes	Coniochaetales	Coniochaetaceae
101	*Nodulisporium* sp.	1	MT586976	KoLRI49212	Sordariomycetes	Xylariales	Xylariaceae
102	*Teichospora* sp.	5	MT586977	KoLRI49482	Dothideomycetes	Pleosporales	Teichosporaceae
103	Dothideomycetes sp.	4	MT586978	KoLRI49023	Dothideomycetes	Dothideomycetes_Un[5]	Dothideomycetes_Un[5]
104	*Phanerochaete concrescens*	7	MT586979	KoLRI49471	Agaricomycetes	Polyporales	Phanerochaetaceae
	104 species (in total)	1885 isolates (in total)					

NI[1], number of isolates; The accession numbers of GenBank[2] and the isolation numbers[3] were represented by one strain of each species; Inc[4], Incertae sedis; Un[5], unidentified.

Table S2. Number of ELF species belonging to different orders in the ELF communities isolated from different isolation conditions.

Class	Order / Variable	Part			Sterilization			Size			Media			
		Center	Middle[2]	Margin	Mild	Moderate[2]	Severe	Small[2]	Medium	Large	BBM	LB	MY	PDA[2]
Agaricomycetes	Agaricales	2	2	0	3	2	0	2	0	0	2	0	1	2
	Cantharellales	2	1	0	3	1	0	1	0	0	1	0	1	1
	Corticiales	1	1	0	0	1	0	1	0	0	3	0	1	1
	Hymenochaetales	4	5	2	0	5	0	5	0	0	0	0	3	5
	Polyporales	26	15	7	25	15	1	15	0	0	8	4	7	15
	Russulales	1	10	3	3	10	0	10	0	0	0	0	1	10
Dothideomycetes	Capnodiales	2	2	5	2	2	0	2	0	0	4	1	0	2
	Dothideomycetes_Un[1]	0	1	0	0	1	0	1	0	0	3	0	0	1
	Pleosporales	1	4	1	1	4	0	4	0	0	5	4	1	4
Eurotiomycetes	Chaetothyriales	1	1	1	0	1	4	1	0	0	26	0	15	1
	Eurotiales	1	7	0	6	7	5	7	0	0	1	4	8	7
Leotiomycetes	Helotiales	0	0	1	0	0	0	0	0	0	1	0	0	0
Pezizomycetes	Pezizales	1	1	1	1	1	0	1	0	0	0	0	0	1
Sordariomycetes	Coniochaetales	2	3	4	5	3	1	3	0	0	5	4	11	3
	Diaporthales	0	0	0	4	0	0	0	0	0	0	0	0	0
	Hypocreales	25	69	43	83	69	43	69	12	11	43	54	96	69
	Microascales	7	15	11	5	15	11	15	0	0	1	8	1	15
	Sordariales	13	25	1	0	25	3	25	4	1	8	12	11	25
	Togniniales	1	1	0	0	1	0	1	0	0	1	0	0	1
	Trichosphaeriales	14	16	20	10	16	1	16	16	13	1	15	3	16
	Xylariales	141	149	71	153	149	69	149	24	28	71	38	102	149
Total		245	328	171	304	328	138	328	56	53	184	144	262	328

Un[1] :Unidentified; Middle[2]-Moderate[2]-Small[2]-PDA[2] are the same data as a control. All data were obtained under combined condition. For example, Center (in Part) represents the combined condition of Center-Moderate-Small-PDA.

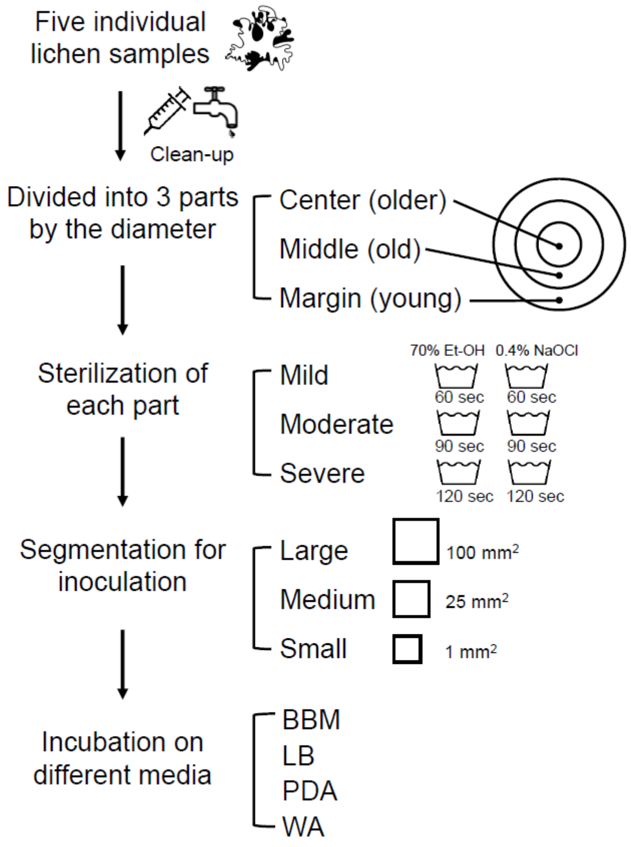

Figure S1. Schematicdiagramofexperimentaldesignadoptedfor ELFisoaltion.

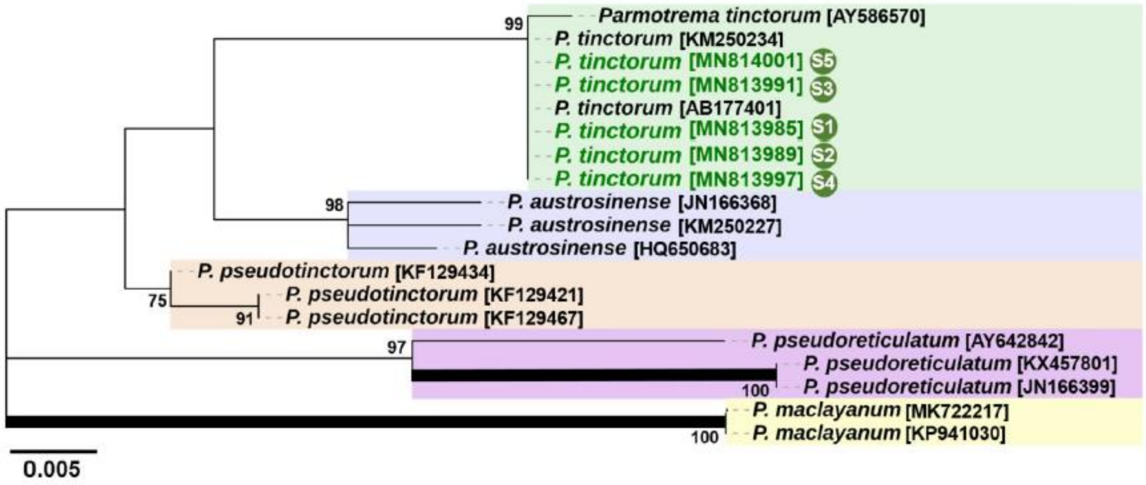

Figure S2. Phylogenetic identification of host lichen *Parmotrema tinctorum*. Maximum likelihood (ML) phylogenetic tree of the ITS nrDNA gene showed phylogenetic relationships of five species (Each species is marked with a different color range.) of *Parmotrema*. The samples used in this study are marked with green color. The site numbers are written next to the names of samples (S1,S2,S3,S4,S5). Bootstrap (BS) values (1000replicates) > 70% of ML are shown near the nodes. Branches supported by BS = 100% are highlighted with thick lines.

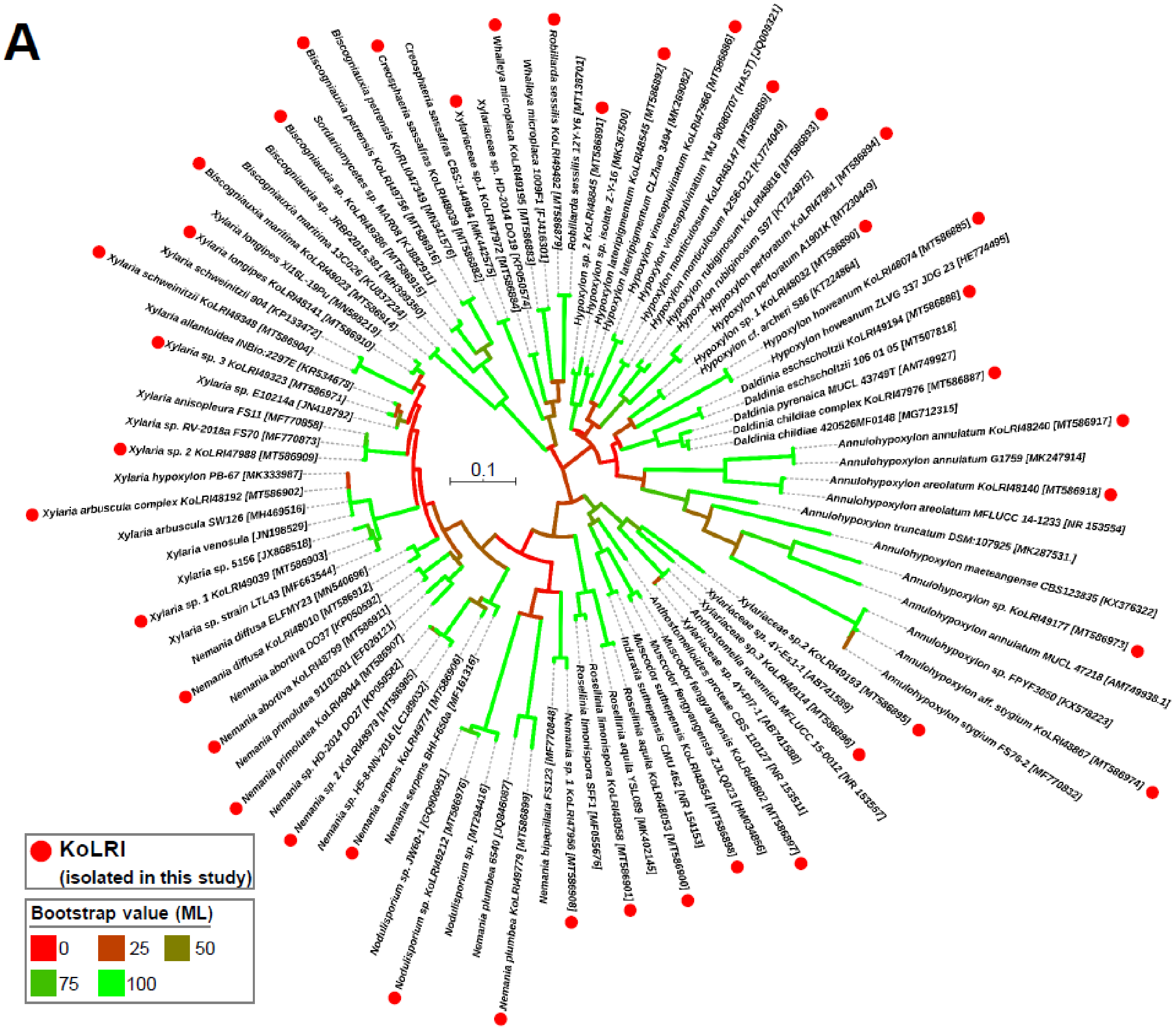
A
0.1
KoLRI
(isolated in this study)
Bootstrap value (ML)
0
25
50
75
100
Annulohypoxylon annulatum KoLRI48240 [MT586917]
Annulohypoxylon annulatum G1759 [MK247914]
Annulohypoxylon areolatum KoLRI48140 [MT586918]
Annulohypoxylon areolatum MFLUCC 14-1233 [NR 153554]
Annulohypoxylon truncatum DSM:107925 [MK287531.]
Annulohypoxylon maeteangense CBS123835 [KX376322]
Annulohypoxylon sp. KoLRI49177 [MT586973]
Annulohypoxylon annulatum MUCL 47218 [AM749938.1]
Annulohypoxylon sp. FPYF3050 [KX578223]
Annulohypoxylon aff. stygium KoLRI48867 [MT586974]
Annulohypoxylon stygium FS76-2 [MF770832]
Xylaria hypoxylon PB-67 [MK333987]
Xylaria arbuscula complex KoLRI48192 [MT586902]
Xylaria arbuscula SW126 [MH469516]
Xylaria venosula [JN198529]
Xylaria sp. 5156 [JX868518]
Xylaria sp. 1 KoLRI49039 [MT586903]
Xylaria sp. strain LTL43 [MF663544]
Xylaria sp. 2 KoLRI47988 [MT586909]
Xylaria sp. RV-2018a FS70 [MF770873]
Xylaria anisopleura FS11 [MF770858]
Xylaria sp. E10214a [JN418792]
Xylaria sp. 3 KoLRI49323 [MT586971]
Xylaria allantoidea INBio:2297E [KR534678]
Xylaria schweinitzii KoLRI48348 [MT586904]
Xylaria schweinitzii 904 [KP133472]
Xylaria longipes KoLRI48141 [MT586910]
Xylaria longipes XI16L-19PU [MN588219]

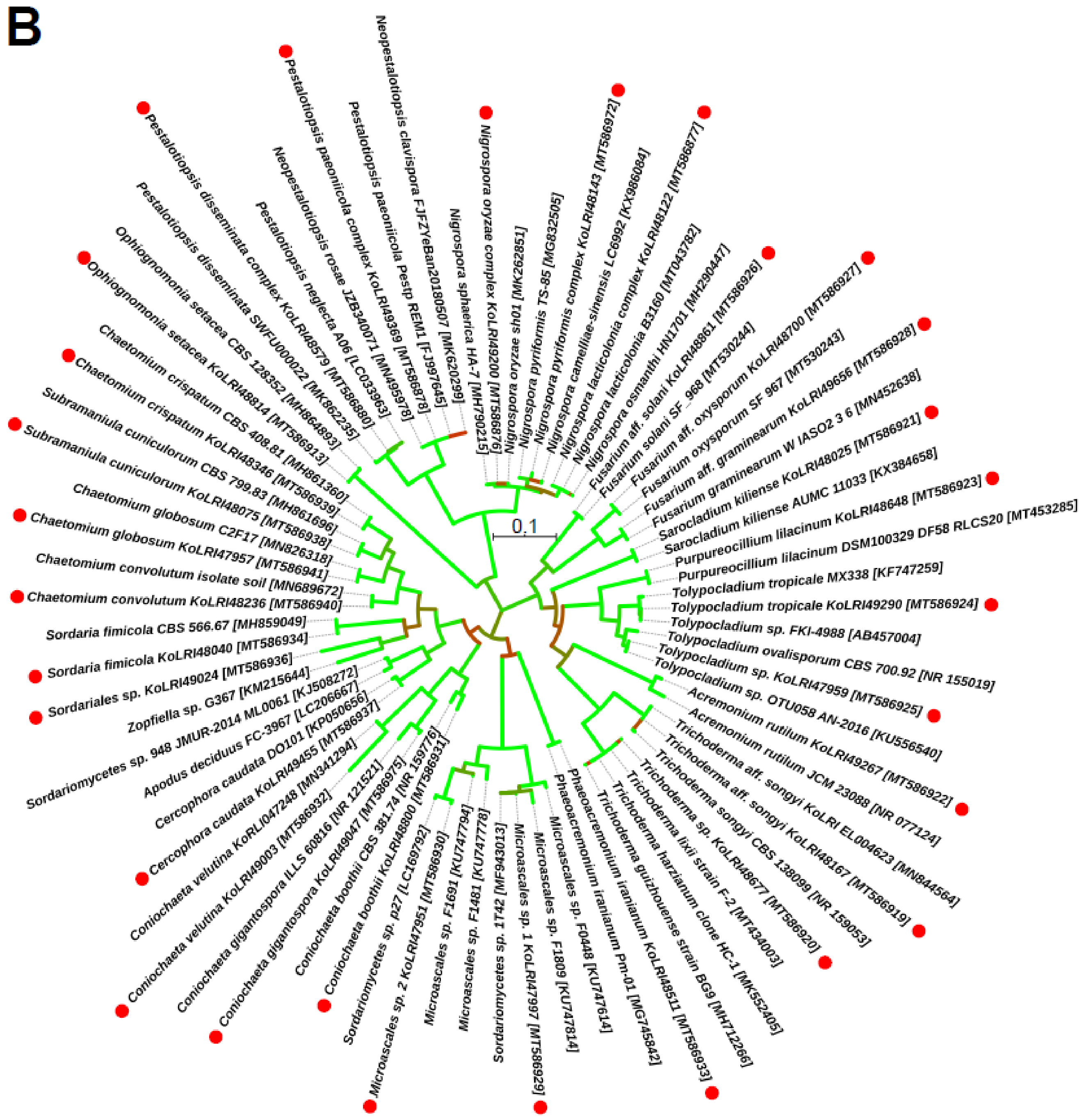

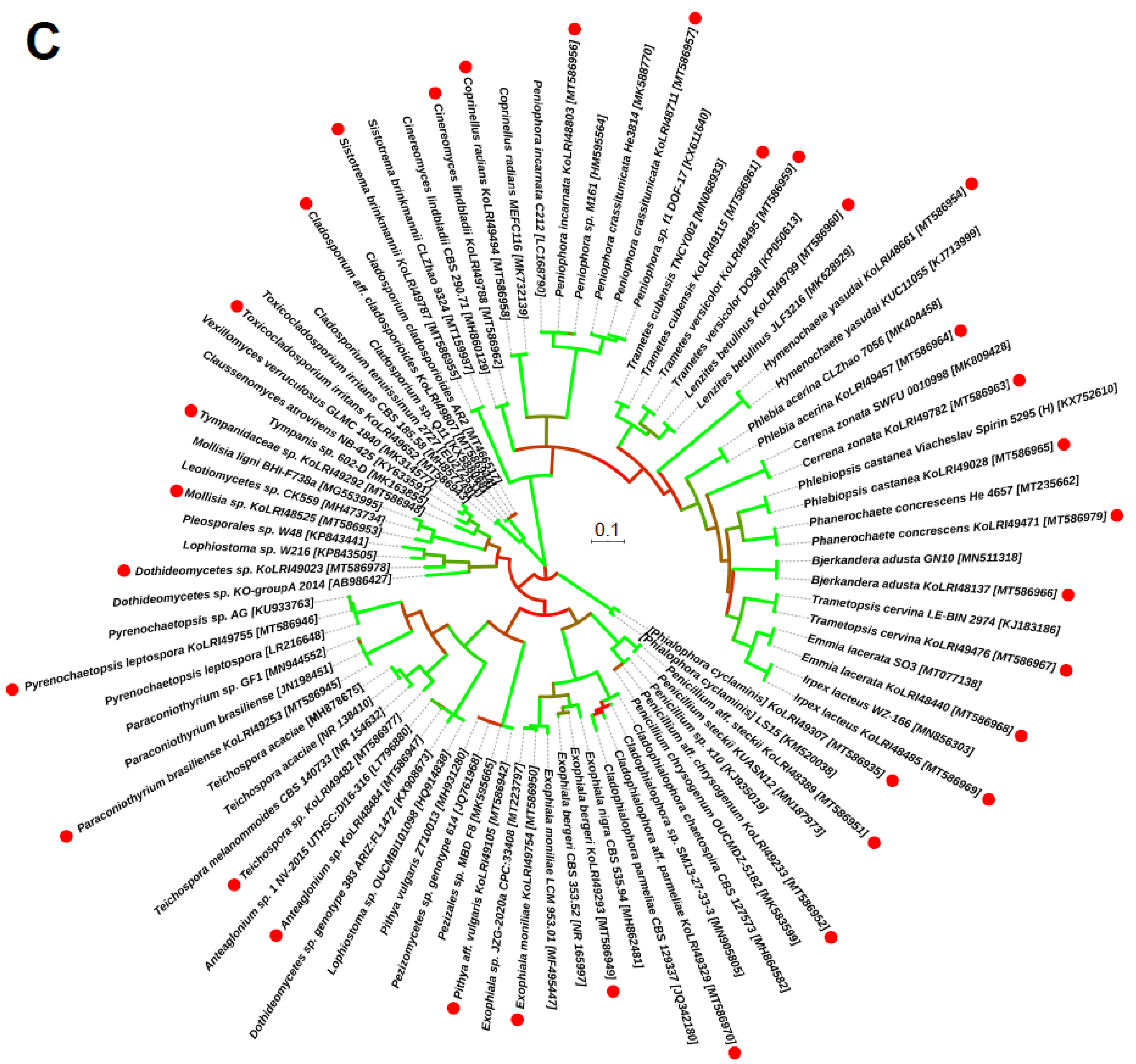

Figure S3. Phylogenetic relationship of isolated ELF. Maximum likelihood (ML) phylogenetic tree of the ITS nrDNA gene showed phylogenetic relationship of isolated ELF: phylogenetic tree of (**A**) Xylariaceae; (**B***)* Sordariomycetes except Xylariaceae; (**C**) Whole taxa except Sordariomycetes. The fungal group isolated in this study are marked with red circles. BS values (1000 replicates) of ML are represented by different colors.

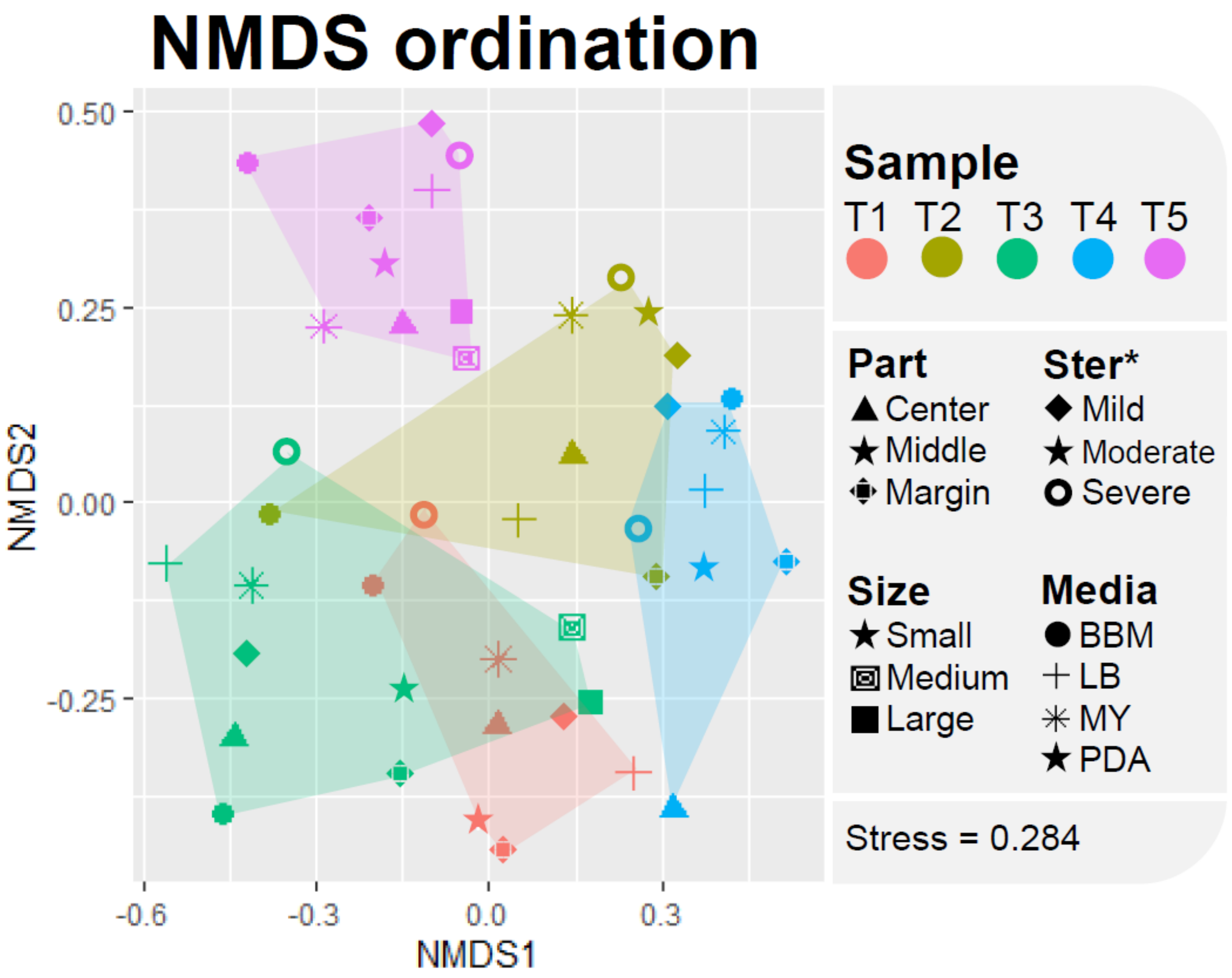

Figure S4. NMDS ordination of isolated ELF community. Each thallus sample is represented in different colors of symbols. The different isolation conditions are represented in different symbols. The stress value is on the right-downside. Ster*, Sterilization.
